# MicroRNA in United Airway Diseases

**DOI:** 10.3390/ijms17050716

**Published:** 2016-05-12

**Authors:** Zheng Liu, Xin-Hao Zhang, Borja Callejas-Díaz, Joaquim Mullol

**Affiliations:** 1 Department of Otolaryngology-Head and Neck Surgery, Tongji Hospital, Tongji Medical College, Huazhong University of Science and Technology, Wuhan 430030, China; zhengliuent@hotmail.com (Z.L.); azhang1@163.com (X.-H.Z.); 2 Rhinology Unit and Smell Clinic, Otorhinolaryngology Department, Hospital Clinic, University of Barcelona & Clinical and Experimental Respiratory Immunoallergy, IDIBAPS, CIBERES, Barcelona 08036, Catalonia, Spain; borch.cd82@gmail.com

**Keywords:** united airways disease, microRNA, inflammation, allergic rhinitis, rhinosinusitis, asthma

## Abstract

The concept of united airway diseases (UAD) has received increasing attention in recent years. Sustained and increased inflammation is a common feature of UAD, which is inevitably accompanied with marked gene modification and tight gene regulation. However, gene regulation in the common inflammatory processes in UAD remains unclear. MicroRNA (miRNA), a novel regulator of gene expression, has been considered to be involved in many inflammatory diseases. Although there are an increasing number of studies of miRNAs in inflammatory upper and lower airway diseases, few miRNAs have been identified that directly link the upper and lower airways. In this article, therefore, we reviewed the relevant studies available in order to improve the understanding of the roles of miRNAs in the interaction and pathogenesis of UAD.

## 1. Introduction

The close link between the upper and lower airways has been carefully investigated in recent years, leading to recognition of united airway diseases (UAD) [1,2]. The evidence showing the link between upper and lower airways first came to light from epidemiological studies, which have shown strong relationships between allergic rhinitis and asthma, as well as between chronic rhinosinusitis (CRS) and asthma or chronic obstructive pulmonary disease (COPD). Approximately 19%–38% of patients with allergic rhinitis (AR) have concomitant asthma, and 30%–80% of asthmatic patients have AR [3]. In a very recent cross-sectional survey of 851 CRS patients in seven cities in China, it was observed that the prevalence of CRS was 24% in asthmatic patients, which was much higher than the 7% in subjects without asthma [4]. The patients with COPD were about two times more likely to develop CRS than those without COPD [4]. In addition, a follow-up study of 99 allergic patients up to 10 years showed that 31.8% of rhinitis sufferers developed asthma, and 50% of asthmatics developed rhinitis [5]. Clinical trials showed that treatment of rhinitis and sinusitis might affect asthma control. Nasal corticosteroids on asthmatic patients reduced emergency department visits and hospitalizations for asthma exacerbations [6,7]. Endoscopic sinus surgery for CRS could improve asthma control significantly and persistently [8].

The link between upper and lower airways also stems from similarities in anatomical structures and functions. The respiratory tract is lined by ciliary epithelium, lamina propria, and glands. The upper airway, especially the nose, functions as a physical filter for the lower airways. Any impairment of this physical filter may result in an alteration of the homeostasis of the lower respiratory airways. For instance, postnasal drip is a possible mechanism causing pulmonary inflammation in patients with rhinitis [2].

Immunological studies have shown that the inflammatory processes in allergic upper and lower airway diseases share overlapping immunopathologic features. In allergic inflammation, the following occur in both upper and lower airways: activation of epithelial immune response, infiltration of eosinophils, IgE production, and activation of mast cells, all of which exaggerate Th2 response [9]. During these processes, several gene expression changes occur, all of which are under tight gene regulation. However, gene regulatory mechanisms underlying common inflammatory processes in UAD remain unclear, creating an obstacle to the better understanding of “one airway, one disease”. Investigation of gene regulation in the processes of inflammation may provide a novel insight into the association of upper and lower airway diseases.

While regulation on the transcriptional level has been a focus of research for a long time, the discovery of gene regulation by small non-coding RNAs has revolutionized the picture of gene expression recently [10,11]. MicroRNA (miRNA), a novel regulator of gene expression, has been considered to be involved in many biological processes, such as cell differentiation, proliferation, and survival [12,13]. miRNAs are identified as an important player in the pathogenesis of many inflammatory diseases including allergy [14,15]. Furthermore, miRNAs may serve as potentially important clinical biomarkers for diagnosis, classification, and outcome prediction of certain diseases [16,17,18]. Indeed, in a very recent article [19], it has been established that subsets of circulating miRNAs are uniquely expressed in patients with AR and asthmatic patients, and have potential for use as noninvasive biomarkers to diagnose and characterize these diseases. Correcting defects in the miRNA regulatory network may represent an alternative and promising approach for nonsteroidal anti-inflammatory treatment [20]. This review summarizes recent advances in the expression and function of miRNAs in UAD in order to promote our understanding of common gene regulation mechanism mediated by miRNAs in UAD.

## 2. MicroRNA Expression in Patients with Coexistence of AR and Asthma

Few studies have investigated the miRNAs that may potentially participate in the pathogenesis of both AR and asthma. Two reports from one laboratory studied miRNA expression profiles in nasal biopsy from patients with a coexistence of AR and asthma [21,22]. In the first study, Suojalehto *et al.* found that 10 miRNAs were differentially expressed in asthmatic patients and controls, but independent of concomitant AR [21]. Asthmatic patients demonstrated increased expression of several miRNAs (miR-143, miR-187, miR-498, miR-874 and miR-886-3p) and decreased expression of other miRNAs (let-7e, miR-18a, miR-126, miR-155 and miR-224) [21] compared with controls, and yet there was no difference in miRNA expression and cellular infiltration between asthmatic patients with and without AR [21]. This is remarkable and unexpected, and may be down to the absence of seasonal allergen exposure during the study. In the second study, the authors measured the inflammatory profile of nasal mucosa in AR patients with and without asthma, and investigated whether this inflammation feature could be modified by asthma [22]. They found that subjects with current AR symptoms had increased levels of miR-155, miR-205, and miR-498, but reduced levels of let-7e in nasal mucosa. Furthermore, patients with positive skin prick test also showed increased expression of miR-155 and miR-205, and decreased let-7a expression as compared to subjects with negative skin prick test [22]. Nevertheless, Th2 cytokines expression levels (IL-4, IL-5 and IL-13) and miRNA expression profile were similar in AR patients with and without asthma, suggesting that concomitant asthma might have a minor impact on the inflammatory profile and miRNA expression of AR patients [22]. However, the onset and severity of asthma were not clearly documented in the study cited. It is possible that the minimal effect of asthma on the inflammation process of AR was due to the mild severity of asthma.

Although those two studies did not demonstrate an obvious interplay between AR and asthma on nasal mucosal miRNA expressions, it may be due to the improper time point of sampling and the mild severity of asthma and AR. When study was conducted out of seasonal allergy exposure or out of an episode of asthma exacerbation, the alterations in miRNA expression might not be easily detectable. Therefore, further studies with careful patient and time point selection would be helpful to clarify the interaction of asthma and AR on the miRNA expression. Moreover, despite total RNA samples being obtained from all frozen nasal biopsies in both studies [21,22], only the expression of a few selected miRNAs was analyzed by quantitative real time PCR (qRT-PCR) in both patient groups and control subjects. It would be interesting to perform a global analysis of the microRNA transcriptome with total RNA, including miRNAs, extracted from tissue samples of patients with AR and asthma.

That notwithstanding, a very recent article [19] has established that a subset of circulating miRNAs are uniquely expressed in patients with AR and asthmatic patients, and have the potential for use as noninvasive biomarkers to diagnose and characterize these diseases. Panganiban *et al.* measured the expression of 420 miRNAs by using qRT-PCR technology in plasma obtained from healthy, AR and asthmatic subjects. They found that 30 miRNAs were differentially expressed among healthy, AR and asthmatic subjects, with those miRNAs being classified into five expression pattern groups based on differences in expression between the asthma and AR, AR and healthy, and asthma and healthy groups. One of those groups showed 10 miRNAs (miR-26b, miR-29, miR-133a, miR-133b, miR-330-5p, miR-144, miR-145, miR-422, miR-1248, and miR-1291) that were concordantly dysregulated by a similar magnitude in patients with AR and asthma. These findings showing a common expression pattern of circulating miRNAs might be reflective of the common pathways involved in AR and asthma. Further studies are therefore needed to validate these promising findings, which suggest that circulating miRNAs have potential for use as non-invasive biomarkers for the diagnosis of UAD, as well as to characterize AR, asthma and other allergic diseases.

## 3. MicroRNAs in Common Inflammatory Processes of UAD

Although upper and lower airway diseases share a lot of common pathways, few studies have investigated the same particular miRNA in the pathophysiology of both AR and asthma. Therefore, we summarized the miRNAs involved in the pathways common to AR and asthma, such as IL-13, GATA binding protein 3 (GATA-3), and mucin secretion, which have been discovered either in asthma or in the study of AR. We did not include the miRNAs involved only in the unique processes of AR or asthma. Those miRNAs involved particularly in AR or in asthma have been well described in recently published reviews [14,23].

**Figure 1:**
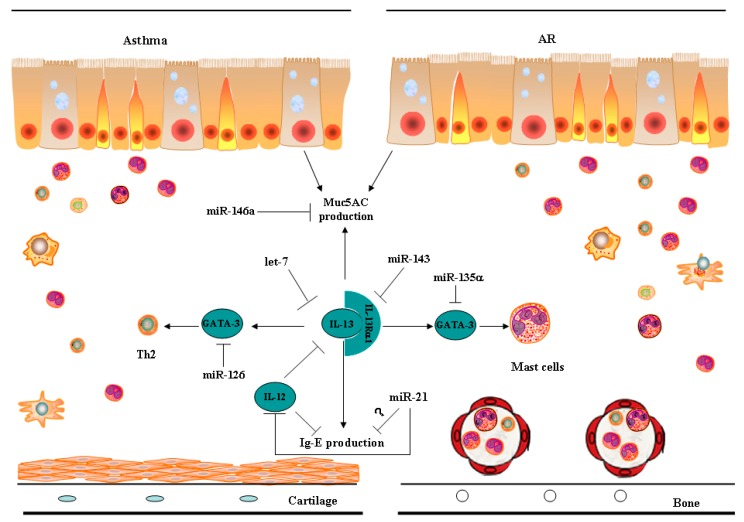
A summary of miRNAs potentially involved in the common pathways in allergic rhinitis and asthma. Effects of induction or stimulation (black arrows), repression or inhibition (T arrows) or indirect repression or inhibition (question mark) are shown.

In one paper concerning miRNA expression in AR, Chen *et al.* screened for the expression of a panel of 157 miRNAs by using RT-PCR in mononuclear leucocytes from human umbilical cord blood (CB) samples and analyzed the association of miRNAs expression and development of AR [24]. They provided the first insight into the role of miR-21 in AR by showing that lower miR-21 expression in CB and increased TGFBR2 expression is associated with antenatal IgE production and development of AR (Figure 1) [24]. Moreover, they found that miR-21 could suppress TGFBR2 expression and a significant increase in TGFBR2 expression in nasal epithelium from AR patients has been reported previously [25]. Chen *et al.* showed that TGFBR2 expression on monocytes was significantly up-regulated in CB with elevated IgE levels and in AR patients. These results indicated the regulatory association between miR-21 and TGFBR2 in AR [24]. In contrast, miR-21 seems to play a different role in the experimental asthma developed in IL-13 transgenic mice [26]. Using a highly sensitive microarray-based approach, Lue *et al.* observed that miR-21 was increased in lung biopsy of IL-13 transgenic mice, with allergic inflammation, and demonstrated that miR-21 is responsible for decreased IL-12 expression, which would partially account for the exaggerated Th2 response seen in asthma (Figure 1) [26]. These findings were validated by another independent model, ovalbumin (OVA)-treated miR-21^−/^^−^ murine model. The miR-21^−/^^−^ mice demonstrated up-regulated levels of IFN-γ and IL-12 compared with wild type mice after OVA treatment [26]. Interestingly, in contrast to studies showing that miR-21 seems to play a different role in AR and asthma, increased levels of circulating miR-21 have recently been found in the plasma of patients with AR and asthma versus healthy control subjects [19]. Therefore, the role of miR-21 in airway allergic diseases needs further investigation.

Interleukin-13 (IL-13), a typical Th2 cytokine, is a central mediator of IgE-mediated allergic diseases. Given its well-established role in modulating Th2 immune responses in AR and asthma, great efforts have been taken to investigate the modulation of IL-13. Using miRNA microarray technology, Shaoqing *et al.* found that miR-143 was the most significantly down-regulated miRNA in nasal mucosal tissues from AR patients compared with controls [27]. Further studies showed that the forced expression of miR-143 significantly decreased the expression of granulocyte-macrophage colony stimulating factor, eotaxin and mucin (Muc) 5AC in nasal epithelial cells after IL-13 stimulation [28]. Importantly, IL-13 receptor α1 chain (IL13Rα1) was identified as a direct target of miR-143 (Figure 1). Therefore, the IL13Rα1 signaling pathway may be a potential target for the prevention and treatment of AR by miR-143 [28]. Likewise, IL-13 was identified as a target of let-7 in an IL-13-dependent murine model of asthma (Figure 1) [20]. Induced levels of IL-13 in cultured T cells were inversely related to let-7 levels. Furthermore, intranasal delivery of let-7 mimic resulted in a decrease in IL-13 levels and alleviated asthma features [20]. Given their ability to regulate IL-13, miR-143 and let-7 may represent potential therapeutic targets for the treatment of UAD.

GATA binding protein 3 (GATA-3) is identified as an important transcription factor of mast cells and Th2 cells, which are key effector cells in the pathogenesis of AR and asthma through releasing inflammatory mediators and cytokines upon exposure to allergens. miRNAs have been shown to regulate the development of mast cells. The forced expression of miR-221 in mast cells led to increased degranulation, cytokine production, and adherence [29]. miR-146a, another miRNA up-regulated upon mast cell activation, contributed to the regulation of cell homeostasis and survival [30]. Nevertheless, the role of miRNA in mast cells in the context of allergic airway diseases has not been thoroughly investigated. Recently, GATA-3 in mast cells has been revealed as a target of miR-135α [31]. Intranasal administration of lentiviral miR-135α in OVA-sensitized AR mice reduced serum IgE concentration and GATA-3 level, as well as eosinophil and mast cell infiltration in submucosal region, all of which favored Th2 response [31]. Laser scanning confocal microscopy demonstrated that miR-135α lentiviral infection specifically targets mast cells (Figure 1) [31]. In the context of asthma, GATA-3 has been found to be modulated by miR-126 in an indirect way. miR-126 blockage resulted in augmented expression of POU (Pit-Oct-Unc) domain class 2 associating factor 1, which activates the transcription factor PU.1 that in turn suppresses Th2 cell function via negative regulation of GATA-3 expression [32]. In allergic mice treated with house dust mite, antagonism of miR-126 decreased Th2 responses, airway hyperresponsiveness, eosinophil recruitment, and mucus hypersecretion [32]. These findings provide evidence that miR-126 contributes to the regulation of allergic airway inflammation by modulating Th2 responses [32]. Until now, the impact of miRNAs on the effector cells in upper and lower airway diseases was studied separately. Additional research is required to confirm the participation of these miRNAs in both AR and asthma.

Goblet cell hyperplasia and mucus hypersecretion are common characteristics of UAD. Muc5AC is recognized as one of the major components of mucus, which is linked to goblet cell hyperplasia. Recent work demonstrated that ectopic expression of miR-146a reduced Muc5AC expression, while depletion of intrinsic miR-146a increased the level of Muc5AC in airway epithelial cells [33]. The favorable effects of miR-146a depletion on Muc5AC expression may be mediated through the activation of JNK and NF-κB signaling (Figure 1) [33]. This is the first report on the regulation of airway mucus production by miRNA. Unfortunately, these findings were only based on *in vitro* data. Further investigations on the therapeutic potential of manipulating miR-146a in the management of mucus overproduction are needed in the context of airway diseases.

Besides the regulation of mucus production, miR-146a was also associated with the clinical efficacy of immunotherapy. In a recently published report, miR-146a expression level in peripheral blood mononuclear cells of 24 AR patients treated with immunotherapy was examined [34]. The results indicated that miR-146a might be modulated by immunotherapy [34]. This discovery highlighted the possibility of miR-146a as a biomarker of immunotherapy [34].

## 4. MicroRNA Profiling in UAD

Technological advances have spawned a multitude of platforms for miRNA profiling [35], and an understanding of the strengths and pitfalls of different approaches can aid in their effective use. In this sense, depending on the tissue sample, it is fundamental to select the most adequate protocol. In extracting miRNAs for instance, given that isolating miRNAs from frozen nasal biopsies [21,22] is not the same as for blood or serum samples [19]. Other important aspects that have to be considered in studying miRNAs are the methods and technology selected for miRNA expression and functional analysis, as well as for data processing, quality assessment and normalization of differential expression levels [35]. As aforementioned, some authors have analyzed the expression of selected miRNAs by quantitative RT-PCR [21,22,24] while others have performed global transcriptomic analysis by microarrays [26,27]. Different strategies were therefore considered depending on the aim of study: to discover novel miRNA candidates; to validate the role of known and well understood miRNA on specific target genes; or to achieve both goals. In summary, methods and technology require particularly careful consideration before future studies on miRNAs in UAD are designed and performed [35]. This pursuit, which is beyond the scope of the current review, will be left to a more specialized article.

## 5. Conclusions

A number of individual miRNAs have been associated with upper or lower airway diseases [36], and emerging results [19] have shown how the expression pattern of circulating miRNAs might have potential for use as non-invasive biomarkers to diagnose and characterize these diseases. However, collaborative study of a particular miRNA in both upper and lower airway diseases simultaneously is lacking. Some results are difficult to explain without careful selection of study time point and study subjects. Moreover, the function network of most miRNAs in airway diseases has not been established. The role of miRNAs in the common pathways of the upper and lower airways and in the interaction of UAD is therefore far from being fully clarified. It is critical to address the following in the future: (1) to identify those miRNAs closely linking the upper and lower airway disease; (2) to validate the potential use of circulating miRNAs as noninvasive biomarkers to diagnose and characterize UAD; and (3) to identify the collaborative roles of miRNAs and the miRNA/mRNA target network in the connection of upper and lower airway diseases. Uncovering the role of miRNAs in UAD may not only contribute to the understanding of the interaction and pathogenesis of UAD, but also provide potential pharmaceutical targets for anti-inflammatory treatment.
